# Control of Postharvest Bacterial Soft Rot by Gamma Irradiation and its Potential Modes of Action

**DOI:** 10.5423/PPJ.NT.08.2015.0165

**Published:** 2016-04-01

**Authors:** Rae-Dong Jeong, Eun-Hee Chu, Duck Hwan Park, Hae-Jun Park

**Affiliations:** 1 Research Division for Biotechnology, Advanced Radiation Technology Institute, Korea Atomic Energy Research Institute, Jeongeup 580-185, Korea; 2 Applied Biology Program, Division of Bioresource Sciences, Kangwon National University, Chuncheon 200-701, Korea

**Keywords:** control, *Erwinia carotovora* subsp. *carotovora*, gamma irradiation, postharvest

## Abstract

Gamma irradiation was evaluated for its *in vitro* and *in vivo* antibacterial activity against a postharvest bacterial pathogen, *Erwinia carotovora* subsp. *carotovora* (*Ecc*). Gamma irradiation in a bacteria cell suspension resulted in a dramatic reduction of the viable counts as well as an increase in the amounts of DNA and protein released from the cells. Gamma irradiation showed complete inactivation of *Ecc*, especially at a dose of 0.6 kGy. In addition, scanning electron microscopy of irradiated cells revealed severe damage on the surface of most bacterial cells. Along with the morphological changes of cells by gamma irradiation, it also affected the membrane integrity in a dose-dependent manner. The mechanisms by which the gamma irradiation decreased the bacterial soft rot can be directly associated with the disruption of the cell membrane of the bacterial pathogen, along with DNA fragmentation, results in dose-dependent cell inactivation. These findings suggest that gamma irradiation has potential as an antibacterial approach to reduce the severity of the soft rot of paprika.

Bacterial soft rot, caused by *Erwinia carotovora* subsp. *carotovora* (*Ecc*), is one of the most important and widespread bacterial diseases of a wide variety of vegetables such as green peppers, paprika, potatoes, and cabbage (Kikumoto, 2000; Bhat et al., 2010). Currently, the primary methods for controlling postharvest bacterial soft rot are bactericides, such as hypochlorite, formaldehyde solution, and antibiotics (Bhat et al., 2010). However, the continued use of chemical bactericides is limited because of the development of bacterial resistance and growing public concerns over the human health and environmental risks over chemical residues. Therefore, it is urgent to develop eco-friendly alternative methods to control postharvest bacterial disease.

In recent years, irradiation has become a viable alternative and an effective nonchemical treatment for control of postharvest pathogens (Hallman, 2011). The exposure of fungi to gamma irradiation triggers chemical and metabolic or physiological changes, which likely block their metabolic functions. The unit of irradiation dose is the gray (Gy). Accumulated evidence shows that gamma irradiation was successfully applied to the control of postharvest pathogens on many fruits, such as *Bacillus cinerea*, *Penicillium italicum*, *Rhizopus stolonifer* var*. stolonifer*, and *Monilinia fructicola* in kiwi (Kim and Yook, 2009). Despite many studies on the control of postharvest fungal pathogens by irradiation, it has not been carried out much for the control of bacterial pathogens and its potential modes of action.

The objective of this study was to examine the effects of gamma irradiation for the control of postharvest bacterial soft rot and its modes of action. We studied the potential mechanism of microbial cell inactivation through the viability of the bacteria, morphological changes, changes in cell membrane integrity, and structural changes in DNA by gamma irradiation. We also suggest a possible new approach of gamma irradiation for controlling the postharvest soft rot on vegetable through an *in vivo* assay.

The inactivation patterns of the gamma-irradiated bacterial cells were investigated using cell suspensions (10^6^ cfu/ml) of *Ecc* (Fig. 1A). The pathogen *Ecc* was donated by Prof. Duck-Hwan Park (Kangwon National University, Korea). *Ecc* was grown overnight in Luria-Bertani (LB) broth at 180 rpm at 28°C. The bacterial cells were harvested, washed, and suspended in 10 mM MgCl_2_. The cells were diluted to a final density of 10^5^ cfu/ml (A_600_) and used for inoculation. A cobalt-60 gamma irradiator at the Korea Atomic Energy Research Institute, Jeongeup, Korea (150 TBq capacity; ACEL, MDS Nordion, Canada) was used for the irradiation. All of the absorbed doses were calibrated using alanine dosimeters with a diameter of 5 mm (Bruker Instruments, Rheinstetten, Germany), where a Bruker EMS 104 EPR analyzer (Bruker Instruments, Rheinstetten, Germany) was used to determine the free-radical signals. The dose uniformity ratio (DUR), as the ratio of the maximal and minimal dose absorbed in the irradiated material (D_max_/D_min_), was approximately one. Survival linear regressions were fitted by plotting the survivor cfu/ml versus the actual radiation doses. The viability of *Ecc* after irradiation revealed that gamma irradiation significantly reduced the viability of bacterial cells. The treatment up to 0.6 kGy resulted in an approximate 4-log reduction of the viable count compared to the initial counts. To further study whether a reduction of cell viability by gamma irradiation is correlated with the cell density, the irradiated-bacteria cell suspensions were serially diluted with 10 mM MgCl_2_ and spread on LB agar plates. The plates were incubated at 28°C for 24 h. Cell density was measured at 600 nm with different time points using a spectrophotometer (Biochrom Libra S70, England). The density of the irradiated cell suspension was dramatically reduced within 6 h of incubation, but in the case of the non-irradiated cell suspension, the density was consistently increased. Therefore, it is assumed that gamma irradiation for bacterial inactivation is highly efficient at a dose of 0.6 kGy. Morphological changes of bacterial cells after irradiation were examined by a scanning electron microscope (SEM). Irradiated-cells were fixed with 3% glutaraldehyde overnight. The samples were dehydrated by successive treatments of ethanol in water; the ethanol concentration for each treatment was increased in concentrations from 50% to 100%. The samples were coated with gold in a sputter coater (JEOL JFC 1100 E; Jeol, Japan) and were examined under a SEM (JEOL, Tokyo, Japan). SEM was employed with cells with irradiation at doses of 0, 0.2, 0.4, and 0.6 kGy, and the shapes of their surface were compared. It was found that non-irradiated cells had a smooth surface, while irradiated-cells appeared more rugged and their surfaces became irregular (Fig. 2). It is obvious from the images that the shapes of higher dose irradiated-cells showed significant structural changes compared to non-irradiated the bacterial cells (Fig. 2). When cells were irradiated at 0.2 kGy, structural changes were not much different. However, a dose of 0.6 kGy showed that irradiated cells showed rough on the cell surface, whereas the non-irradiated cells were short, planiform elliptical rods. These bacteria showed deep surface cracks and a complete decay of the organized structure. The present study demonstrates the remarkable dose-dependent alterations in the surface of the *Ecc* that are caused by gamma irradiation. The damages to the membranes of *Ecc* by gamma irradiation prompted us to examine the membrane integrity. *Ecc* cells were irradiated at different doses in an LB medium. After 2 h of incubation at 28°C, cells were stained with 10 μg/ml propidium iodide (PI) (Sigma-Aldrich) for 5 min at 30°C (Fish et al., 2000). The cells were observed with a Zeiss Axoskop 40 microscope (Carl Zeiss, Oberkochen, Germany) equipped with an individual fluorescein rhodamine filter set (Zeiss no. 15: excitation BP 546/12 nm, Emission 590 nm). The PI was used to determine whether gamma irradiation led to the loss of membrane integrity in *Ecc*. Cells that have lost their membrane integrity showed red staining under a fluorescence microscope. It was shown that, compared with the control, more cells were stained with PI after irradiation (Fig. 3A). This indicates that cells lost their membrane integrity increased after irradiation. Irradiation inhibits the growth of *Ecc* by directly damaging the membrane and causing the cell death of the bacterial pathogen. Damage to the membrane leads to the loss of osmotic balance and an influx of fluids and ions, as well as a loss of proteins and ribonucleic acid, eventually causing cell death (Woo et al., 2000). To verify this hypothesis of whether irradiation caused cell death of *Ecc*, the leakage of the cell materials, nucleic acid and protein, was determined. The amount of nucleic acid and protein released from the irradiated cells was measured at 260 and 595 nm, respectively (Bradford, 1976). All experiments were carried out in triplicate. Nucleic acid and its related compounds, such as pyrimidines and purines, are well known to absorb UV light at a wavelength of 260 nm (Khalil and Villota, 1988). Furthermore, similarly damaged cells are also known to release intracellular proteins into a suspension. The amount of nucleic acid released into the cell suspension was analyzed by measuring the absorbance at 260 nm (Fig. 3B). The amount of leaked nucleic acid from the cells grew relative to increasing doses of the cell suspension. In addition, the amount of protein released into the cell suspension was also analyzed. The amount of leaked protein was shown in a dose-dependent manner. These data suggest that irradiation might directly act on the cell membrane of the bacteria, leading to the breakdown of the cell membrane and release of cell materials. These data verify the destruction cells and provide clear evidence of the antibacterial activity of irradiation. A previous study suggested that e-beam irradiation increases the inactivation of bacteria through DNA fragmentation (Fister et al., 2012). To test this hypothesis, genomic DNA extracted from irradiated cells was measured to determine the effect of gamma irradiation on the DNA integrity. Genomic DNA from the irradiated bacterial cells was extracted using an Exprep plasmid SV mini kit (Gene ALL, Seoul, Korea). The DNA was run on a 1% agarose gel in a 1× TAE buffer. The gels were then stained with ethidium bromide and visualized under UV light. DNA concentrations were standardized at 1.2 μg per well prior to electrophoresis. The increasing dose of GI caused a degradation of the genomic DNA of bacteria (Fig. 4). Smears generally indicate partial fragmentation of the nucleic acid. Oxidative damage to DNA is well studied in *E. coli*, in which hydrogen peroxide generated by irradiation breaks in the DNA sequence. To examine whether gamma irradiation has antibacterial activity *in vivo*, we introduced gamma irradiation to paprika infected with *Ecc* (Fig. 5A). Paprika were disinfected with 1% v/v sodium hypochlorite for 2 min, washed with tap water and dried in air. Paprika was wounded with a sterile nail (one wound per fruit, in the equatorial zone; 2 × 2 mm) and inoculated a cell suspension (10^5^ cell/ml) of *Ecc*. The inoculated paprika were irradiated with 0.1, 0.2, 0.4, and 0.6 kGy of gamma irradiation, and paprika were kept at room temperature (23°C) for 14 days. The complete inhibitory effect was shown in 0.1 kGy (Fig. 5A, B). There were no disease symptoms when irradiated at over a dose of 0.1 kGy (data not shown). A previous study showed that up to 0.4 kGy of gamma irradiation did not affect any physical properties on paprika (Yoon et al., 2014). Consistent with an earlier study, there are no detrimental effects on the quality of the paprika at 0.1 kGy (Song et al., 2014). These results suggest that 0.1 kGy of gamma irradiation efficiently control bacterial soft rot disease of paprika.

In the present study, gamma irradiation can directly inhibit the growth of *Ecc* (*in vitro*) and prevents to disease severity of soft rot on paprika. The reduction of bacterial growth was directly linked to the ability to disrupt the cell membrane and DNA integrity of the bacterial pathogen. Damage leading to membrane rupture and the resulting DNA degradation at the low dose of gamma irradiation appear to have resulted from gamma irradiation-induced free radicals in bacterial cells, although this is yet to be determined. Together, our results allow the development of irradiation decontamination systems at an industrial level. Gamma irradiation is a promising approach for the utilization of synthetic chemicals or pesticides on vegetables. In addition, irradiation has potential use in the control of other bacterial pathogens.

## Figures and Tables

**Fig. 1:**
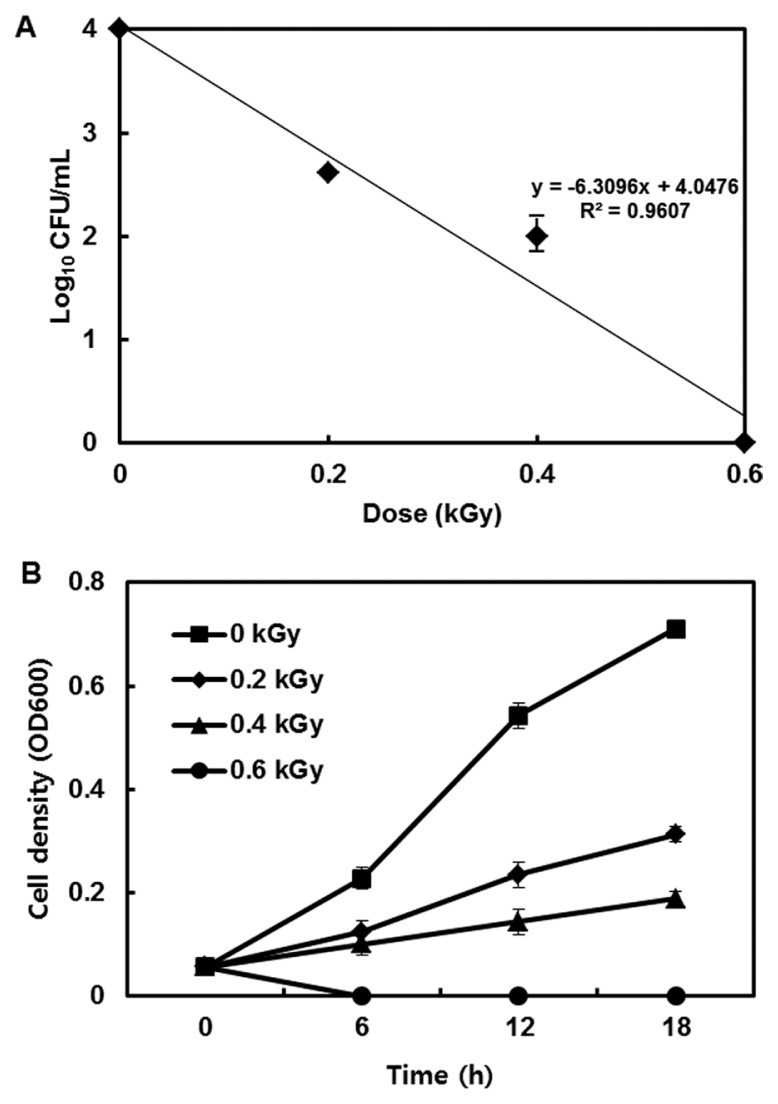
Inhibitory effect of gamma irradiation on *Erwinia carotovora* subsp. *carotovora*. (a) Survival curves of *Ecc*. (b) Sensitivity of non-irradiated and irradiated bacterial cells. Cell density was observed after 18 h incubation after GI. Experiments were conducted independently three times. Error bar is standard errors of three replications.

**Fig. 2:**
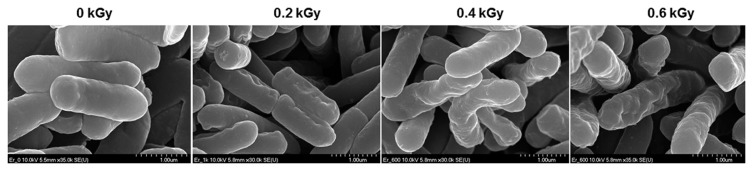
Scanning electron microscope illustrating the effects of gamma irradiation on the morphology of *Ecc*. Microscopy image of *Ecc* after gamma irradiation (0, 0.2, 0.4, and 0.6 kGy). The increasing doses of gamma irradiation exhibited rough on the cell surface.

**Fig. 3:**
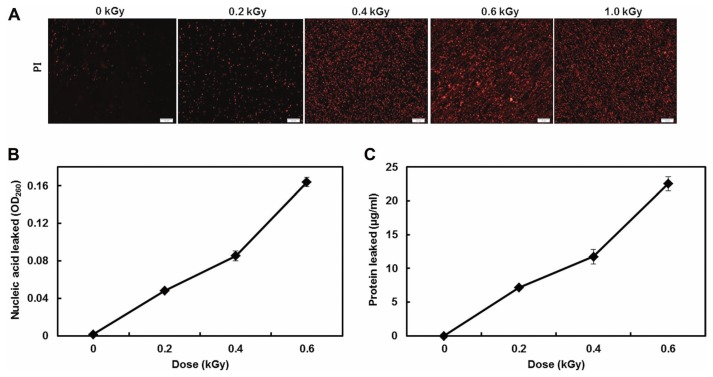
*Ecc* viability and leakage of nucleic acid and protein from irradiated bacterial cells. (A) Loss of membrane integrity of *Ecc* after GI. Bar represents 10 μM. The released amount of nucleic acid (B) and protein (C) in the supernatant of the cell suspension were measured. Error bar is standard errors of three replications.

**Fig. 4:**
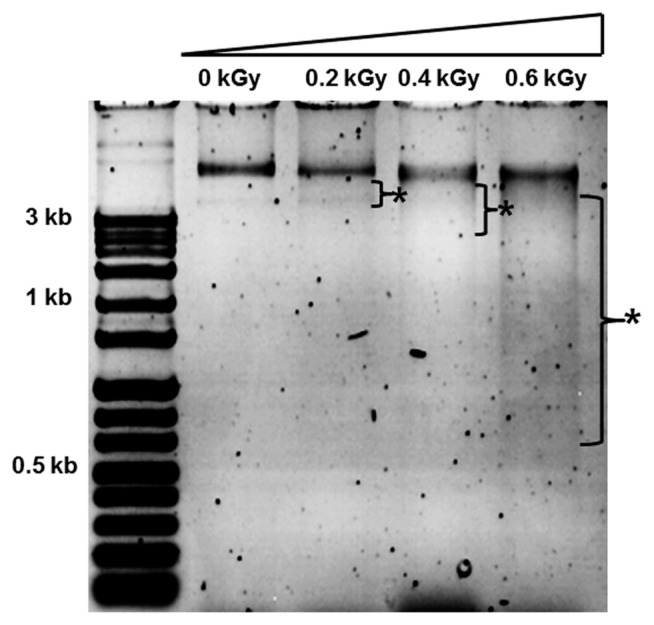
Effect of gamma irradiation on the genomic DNA of bacteria. Visualization of the effect of GI on the electrophoretic pattern of DNA extracted from 0, 0.2, 0.4, and 0.6 kGy. Asterisk indicates smeared DNA in different doses.

**Fig. 5:**
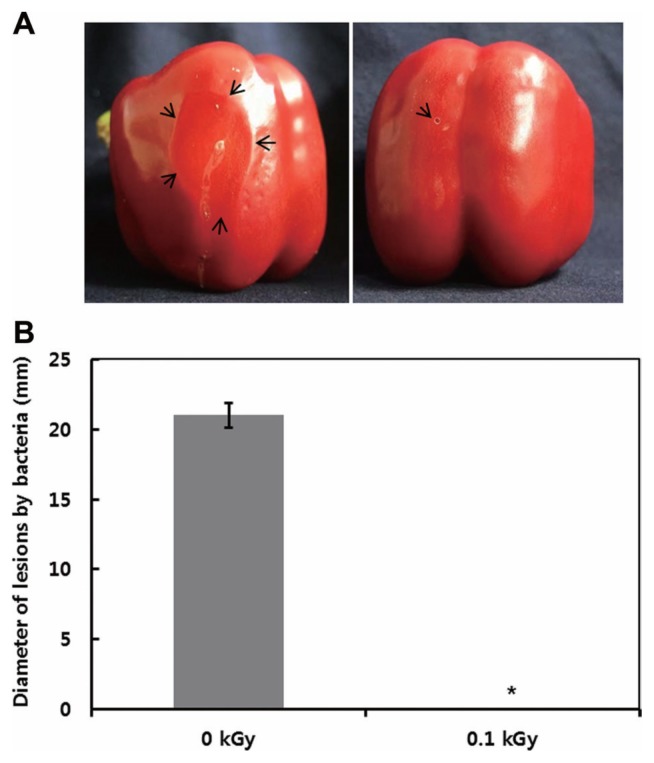
Effect of gamma irradiation on *Ecc* of papriak. Paprika were inoculated with *Ecc* and then irradiated to the above indicated conditions. (a) Size of lesion formed in paprika. Photographs were taken 10 days post-inoculation and (b) lesion diameter was analyzed. Error bars represent the standard error from three independent experiments. Asterisk indicates data statistically significant from that of control (0 kGy) (*P* < 0.05, n=10).
